# Physical and Antimicrobial Properties of Starch-PVA Blend Films as Affected by the Incorporation of Natural Antimicrobial Agents

**DOI:** 10.3390/foods5010003

**Published:** 2015-12-26

**Authors:** Amalia Cano, Maite Cháfer, Amparo Chiralt, Chelo González-Martínez

**Affiliations:** Institute of Food Engineering for the Development, Polytechnic University of Valencia, Camino de Vera s/n, 48022 Valencia, Spain; mtchafer@tal.upv.es (M.C.); dchiratld@tal.upv.es (A.C.); cgonza@tal.upv.es (C.G.-M.)

**Keywords:** oregano essential oil, neem oil, water vapour permeability, mechanical properties, *E. coli*, *L. innocua*, *A. niger*, *P. expansum*

## Abstract

In this work, active films based on starch and PVA (S:PVA ratio of 2:1) were developed by incorporating neem (NO) and oregano essential oils (OEO). First, a screening of the antifungal effectiveness of different natural extracts (echinacea, horsetail extract, liquid smoke and neem seed oil) against two fungus (*P. expansum* and *A. niger*) was carried out. The effect of NO and OEO incorporation on the films’ physical and antimicrobial properties was analyzed. Only composite films containing OEO exhibited antibacterial and antifungal activity. Antibacterial activity occurred at low OEO concentration (6.7%), while antifungal effect required higher doses of OEO in the films. Incorporation of oils did not notably affect the water sorption capacity and water vapor barrier properties of S-PVA films, but reduced their transparency and gloss, especially at the highest concentrations. The mechanical response of the S-PVA films was also negatively affected by oil incorporation but this was only relevant at the highest oil ratio (22%). S-PVA films with 6.7% of OEO exhibited the best physical properties, without significant differences with respect to the S-PVA matrix, while exhibiting antibacterial activity. Thus, the use of OEO as a natural antimicrobial incorporated into starch-PVA films represents a good and novel alternative in food packaging applications.

## 1. Introduction

In the last few years, consumer demand for natural ingredients and foods without synthetic preservatives has increased the popularity of natural antimicrobial agents. So far, many studies have been carried out in order to take advantage of the antibacterial and/or antioxidant activities of natural substances from different sources such as microorganisms, animals and plants [1].

Extracts from natural sources have been used against food spoilage since ancient times to extend food shelf-life and to prevent foodborne diseases. Substances such as alkaloids, tannins, flavonoids and phenolic compounds that are found in plant extracts are responsible for bioactivity [2,3]. These substances have been widely used as food flavoring agents and most of them are generally recognized as safe (GRAS) by the Food and Drug Administration (FDA). In Europe, extracts from natural resources are regulated under Regulation EU 872/2012 that contains the list of flavoring substances authorized for food uses.

Among bioactive plant extracts, it is well known that those from Echinacea (*Echinacea purpurea*), field horsetail (*Equisetum*), neem (*Azadirachta indica*) or essential oils have shown antimicrobial activity against foodborne pathogens or inhibited food spoilage. Significant antimicrobial activity has been attributed to Echinacea extracts, in a series of *in vitro* tests, against *Saccharomyces cerevisiae,* various *Candida* species, *Listeria monocytogenes* and *Staphylococcus* [4,5]. Field horsetail has been also described as an herb with antioxidant and antimicrobial properties. Some studies revealed its inhibitory effect on the *Aspergillus* spp. and *Fusarium* spp. growth and toxin production [6,7,8]. Garcia *et al.* [9] confirmed that a hydro-alcoholic extract of *E. arvense* inhibited the growth of *Aspergillus flavus* and *Fusarium verticillioides* in maize seeds, especially at high water activity levels (simulating pre-harvest conditions).

Neem is also a non-toxic plant which possesses excellent antimicrobial properties [10,11,12]. In fact, Baswa *et al.* [13] revealed that the neem oil has bactericidal activity against 14 strains of pathogenic bacteria such as *Staphylococcus aureus* [14], *Staphylococcus typhus* [15], and *Escherichia coli*, *Streptococcus mutans* and *lactobacilli* [16]. On the other hand, Mahfuzul Hoque *et al.* [17] determined the antibacterial activity of neem extracts against 21 strains of foodborne pathogens: *Listeria monocytogenes*, *Staphylococcus aureus*, *Escherichia coli* O157:H7, *Salmonella* *Enteritidis*, *Vibrio parahaemolyticus*, and *Bacillus cereus*, and five food spoilage bacteria: *Pseudomonas aeroginosa*, *Psuedomas putida*, *Alcaligenes faecalis*, and *Aeromonas hydrophila*. They concluded that neem extracts generally showed higher antimicrobial activity against Gram-positive bacteria than against Gram-negative, and none of the extracts showed antimicrobial activity against *E. coli* O157:H7 and *Salmonella Enteritidis*. The mechanism of action of the neem extracts is mainly attributed to the inhibition of cell-membrane synthesis in the bacteria [13].

Nevertheless, the most widely used extracts from natural sources are essential oils which have exhibited antimicrobial activity against a wide spectrum of bacteria and fungi. They are constituted by hydrophobic, volatile compounds with low molecular weight [18]. Among them, the oregano essential oil is one of the most effective antimicrobial oils and its active properties have been demonstrated in numerous studies [19,20,21]. These have been mainly attributed to carvacrol, thymol, γ-therpinene and p-cymene [22,23,24]. The mode of action of the major components, carvacrol and thymol, as explained by Burt [22], consists of the disintegration of the outer cell membranes of bacteria, releasing lipopolysaccharides and increasing the permeability of the cytoplasmic membrane to ATP. Some authors reported that gram-positives bacteria are slightly more sensitive to the essential oil action than gram-negatives, according to the described mechanism [22,25,26]. 

Another natural antimicrobial agent is the traditional wood smoke that has been used for centuries to preserve food quality on the basis of its antioxidant and antimicrobial properties [27,28]. The antimicrobial properties of the pyrolysis condensate or “liquid smoke” from different woods, with different levels of phenols, carbonyl compounds and organic acids, against *Staphylococcus aureus*, *Aeromonas hydrophila, Salmonella*, *Listeria monocytogenes* and *Escherichia coli* have been recently studied [27].

In regards to the incorporation of antimicrobial agents into food systems, a new concept has gained increasing acceptance in recent years, which is the incorporation of bioactive natural extracts in food packaging, thus obtaining active packaging materials [24]. In this sense, the use of active coatings in postharvest or minimally processed fruits and vegetables, cheeses, meats, *etc.* or the development of bioactive films for food packaging is one of the reasons for the recent gain in importance of natural bioactive substances.

Incorporation of bioactive substances in food coatings or packaging films shows some advantages, including that the compounds are not directly exposed to external conditions [29], they can act only at the surface level and can be applied at any stage of the food supply chain [30,31]. Thus, natural agents have been incorporated into a wide spectrum of natural and synthetic polymer matrices to obtain active materials [32], although no previous studies about the incorporation of bioactive substances into starch-PVA blends has been found, despite that recent studies reported different benefits of this blend film in terms of water vapor barrier and mechanical properties. Starch-PVA films were much more extensible and stable throughout storage and exhibit lower water sorption capacity than pure starch films [33]. However, the incorporation of antimicrobial substances can affect the film properties which are relevant for a specific target application, such as barrier capacity to water vapour, oxygen, CO_2_ or aroma compounds and mechanical and optical properties [34].

The aim of this work was to obtain bioactive S-PVA films for extending the food shelf life by controlling the microbial spoilage. To this end, the antifungal activity of different natural compounds (Echinacea and horsetail extracts, liquid smoke and neem seed oil) against two fungus (*P. expansum* and *A. niger*) was tested at different concentrations. Afterwards, on the basis of the obtained, and previously reported, results, neem oil and oregano essential were incorporated into S-PVA films to analyze their effect on the barrier, optical and mechanical properties of the films as well as the film antimicrobial activity against two fungus, *P. expansum* and *A. niger* and two bacteria, *L. Innocua* and *E. Coli*.

## 2. Materials and Methods

### 2.1. Materials

Pea starch (S) was purchased from Roquette Laisa España S.A. (Benifaió, Valencia, Spain), poly (vinyl alcohol) (PVA) (M_w_: 89,000–98,000, degree of hydrolysis >99%, and viscosity at 4% H_2_O, 20 °C is 11.6–15.4 cP) was obtained from Sigma Aldrich Química S.L. (Madrid, Spain) and glycerol and magnesium nitrate-6-hydrate were provided by Panreac Química S.A. (Castellar de Vallès, Barcelona, Spain).

Different natural antimicrobial substances used were: echinacea (E) and horsetail extract (HS) extract from Soria Natural S.A. (Lérida, Spain), liquid smoke (LS), provided by G. Mariani & C. S.p.a. (Cellatica, Italy), neem oil (NO) purchased from Magnolia Holland Ibérica S.A. (Vilassar de Mar, Barcelona, Spain) and oregano essential oil (OEO) from Herbes del Molí (Benimarfull, Alicante, Spain).

Stock culture of *Escherichia colli* (CECT 515), *Listeria innocua* (CECT 910), and *Asperguillus niger* (CECT 20156) supplied by Colección Española de Cultivos Tipos (CECT, Burjassot, Spain) were kept frozen (−25 °C) respectively in Tryptone Soy Broth (TSB, Scharlab, Barcelona, Spain) for bacteria and Potato Dextrose Broth (Scharlab, Barcelona, Spain) for fungus, supplemented with 30% glycerol. *Penicillium expansum* was provided from the culture collection of Department of Biotechnology (Universitat Politècnica de València, Valencia, Spain).

### 2.2. Preparation of Film Forming Dispersion and Films

Films were obtained by solvent casting procedure after the preparation of the corresponding film forming dispersions (FFDs). First, starch (2% *w*/*w*) was dispersed and heated in an aqueous solution at 95 °C for 30 min to induce starch gelatinization. Thereafter, the dispersion was homogenized using a rotor-stator homogenizer (Ultraturrax D125, Janke and Kunkel, Germany) at 13,500 rpm for 1 min and 20,500 rpm for 3 min. Afterwards, PVA was incorporated to the pregelatinized starch dispersion in a S:PVA ratio of 2:1, and heated, while stirred, at 95 °C for 30 min until complete dissolution. Finally, glycerol was added at a starch:glycerol ratio of 1:0.25, on the basis of previous studies [33]. This FFD was used to obtain the control films (S-PVA) and was also used to incorporate the different antimicrobial substances: oregano essential oil (OEO) or neem oil (NO). These were incorporated into the films at two different ratios with respect to the starch, 1:0.125 (S-PVA-1OEO and S-PVA-1NO) and 1:0.5 (S-PVA-2OEO, S-PVA-2NO), which corresponds to 6.7 and 22 g/100 g total solids in the film, respectively. Afterwards, The FFD was homogenized at 12,500 rpm for 4 min to disperse the lipids.

Controlled volumes of film-forming dispersions (equivalent to 1.5 g of total solids) were cast into levelled Teflon casting plates (15 cm diameter) and dried at 25 °C and 45% RH for 48 h. Then, they were peeled intact from the plates and were conditioned at 53% RH using magnesium nitrate-6-hydrate saturated solution at 25 °C until further analysis.

### 2.3. Physical Properties of Films

#### 2.3.1. Film Thickness

Thickness of the films was measured at six random positions with a Palmer digital micrometer to the nearest 0.0025 mm.

#### 2.3.2. Moisture Content

The moisture content of the films (MC), equilibrated at 53% RH and 25 °C for one and five weeks, was analysed by drying the samples in a vacuum oven at 60 °C for 24 h. Later on, the pre-dried samples were placed in desiccators containing P_2_O_5_ until reaching constant weight. Five replicates per film formulation were considered.

#### 2.3.3. Water Vapour Permeability

Water vapour permeability (WVP) was evaluated in the films equilibrated at 25 °C and 53% RH after one and five storage weeks, following the ASTM E96-95 gravimetric method [35] by using Payne permeability cups (Payne, elcometer SPRL, Hermelle/sd Argenteau, Belgium) 3.5 cm in diameter. The temperature was 25 °C and the relative humidity gradient was 53%–100%, which was obtained using magnesium nitrate-6-hydrate and pure water, respectively. Cups were introduced into desiccators and these into a temperature-controlled chamber at 25 °C. Weight control of the cups was performed every 2 h using an analytical balance (±0.00001 g). The water vapour transmission (WVTR) was determined from the slope obtained from the regression analysis of weight loss data *versus* time, once the steady state had been reached, divided by the film area. Five replications were carried out for each type of film.

#### 2.3.4. Internal Transmittance

The transparency was determined by applying the Kubelka–Munk theory for multiple scattering to the reflection spectra obtained in a spectrocolorimeter CM-3600d (Minolta Co., Tokyo, Japan) with a 30 mm illuminated sample area. This theory assumes that each light flux which passes through the film is partially absorbed and scattered, which is quantified by the absorption (K) and the scattering (S) coefficients. Transparency (K/S) was calculated, as indicated by Hutchings [36], from the reflectance of the sample layer on a white background of known reflectance and on an ideal black background. Measurements were taken triplicate in samples equilibrated at 25 °C and 53% RH for one and five weeks, using both a white and a black background.

#### 2.3.5. Gloss

Gloss was measured using a flat surface gloss meter (Multi-Gloss 268, Minolta, Langenhagen, Germany) at an angle of 60° with respect to the normal to the film surface, according to the ASTM standard D523 [37]. Prior to gloss measurements, films were conditioned at 25 °C and 53% RH for one and five weeks. Gloss measurements were carried out over a black matte standard plate and were taken in triplicate. Results were expressed as gloss units, relative to a highly polished surface of standard black glass with a value close to 100.

#### 2.3.6. Mechanical Properties

Mechanical properties were measured with a Universal Test Machine (TA.XT plus, Stable Micro Systems, Haslemere, UK) following the ASTM standard method D882 [38]. Equilibrated film (25 °C for 1 and 5 weeks at 53% RH) specimens (2.5 cm wide and 10 cm long) were mounted in the film-extension grips (A/TG model) which were set 50 mm apart. The speed of the testing machine during stretching was 50 mm·min^−1^ until breaking. Force-distance curves were obtained and transformed into Stress-Hencky curves which allowed tensile strength at break (TS, MPa), percentage of elongation at break (E, %) and elastic modulus (EM, MPa) to be obtained. Eight samples per formulation were measured.

### 2.4. Microbiological Analysis

#### 2.4.1. Screening Test of the Antifungal Natural Substances

For the screening test, samples of the potentially bioactive substances were introduced in tubes with Potato Dextrose Broth-PDB (Scharlab S.L., Barcelona, Spain) at two concentrations, 1% and 10% (mL substance/100 mL PDB). Immediately after, each tube were inoculated with the inoculum at 10^5^ spores per mL for both *Aspergillus niger* and *Penicillium expansum*, previously sporulated on Potato Dextrose Agar (PDA) at 25 °C. The inoculums’ concentration was adjusted by means of a haemocytometer. As control samples, tubes without antimicrobial substance were considered. After 24 h of incubation, a count of colonies was performed in triplicate. To this end, the tube content was extended on petri dishes (Sterilin Limited, Gwent, UK) with PDA (Scharlab S.L., Barcelona, Spain) and incubated for 5 days at 25 °C.

#### 2.4.2. Antimicrobial Effectiveness of the Films

*In vitro* analysis of the antimicrobial effectiveness of films was carried out by a method adapted from Kristo, *et al.* [39] and Sánchez-González, *et al.* [34] by using two fungus, *Aspergillus niger* and *Penicillium italicum*, and two bacterias, *Listeria innocua* as Gram+ bacteria and *Escherichia coli* as Gram−.

Bacteria were regenerated by transferring a loopful of bacteria into 10 mL of TSB and incubating at 37 °C overnight. A 10 μL aliquot from the overnight culture was again transferred to 10 mL of TSB and grown at 37 °C to the end of the exponential phase of growth. This culture, appropriately diluted, was then used for inoculation of the agar plates in order to obtain a target inoculums of 10^2^ UFC/cm^2^. Tryptone soy agar with 3% NaCl (Panreac química, S.A., Castellar del Vallés, Barcelona, Spain) was used as a model solid food system (TSA-NaCl). Aliquots of TSA-NaCl (20 g) were poured into Petri dishes. After the culture medium solidified, properly diluted overnight culture of each bacteria was inoculated on the surface.

On the other hand, fungi were inoculated on potato dextrose agar (PDA) and incubated at 25 °C until sporulation. The cells were counted in a haemocytometer and diluted to a concentration of 10^5^ spores per mL. Aliquots of PDA (20 g) were poured into Petri dishes. After the culture medium solidified, diluted spore solution of each fungus was inoculated on the surface.

The different tested films of the same diameter as the Petri dishes (containing or not antimicrobial substance) were placed on the inoculated surface. Inoculated and uncoated Petri dishes were used as control in the respective culture medium, for bacteria or fungi. Plates were then covered with parafilm to avoid dehydration and stored for 12 days at 25 °C for fungi and 10 °C for bacteria strains. Selected temperatures tried to simulate practical conditions of application. Microbial counts on plates were carried out immediately after the inoculation and periodically during the storage period (0, 3, 5, 7, 10 and 12 days).

To this end, the agar was removed aseptically from Petri dishes and placed in a sterile fitter stomacher bag (Seward, West Sussex, United Kingdom) with 100 mL of tryptone phosphate water (Sharlab S.A., Barcelona, Spain). The bag was homogenized for 2 min in a Stomacher blender (Bag Mixer 400, Interscience, France). Afterward serial dilutions were prepared and poured onto plates with selective microbial medium. PDA plates were used to obtain the fungus counts while a selective microbial medium was used for bacteria for obtain high selectivity and good colonies. *E. coli* was counted in Violet Red Bilis agar (Sharlab S.A., Barcelona, Spain) plates and in the case of *L. Innocua* in Palcam Agar Base (Sharlab S.A., Barcelona, Spain) supplemented with Palcam Selective Supplement (Sharlab S.A., Barcelona, Spain). Then an incubation of 5 days at 25 °C for fungi and 24 or 48 h at 37 °C for Listeria or *E. coli*, respectively, was carried out. All microbial counts were performed in triplicate.

### 2.5. Statistical Analysis

Statgraphics Centurion XV.I (Manugistics Corp., Rockville, MD, USA) was used for carry out the statistical analysis of results through analysis of variance (ANOVA). To differentiate samples, Fisher’s least significant difference (LSD) was used at the 95% confidence level.

## 3. Results and Discussion

### 3.1. Screening Test: Selection of Antimicrobials

Table 1 shows the viable cell counts obtained for *Aspergillus niger* and *Penicillium expansum* after 24 h in contact with the different extracts at the two different concentrations (1% *v*/*v* and 10% *v*/*v*) in PDB liquid culture. Only neem oil at 10% showed a significant antifungal effect compared with the control sample.

**Table 1:** Viable counts of *Aspergillus niger* and *Penicillium expansum* after 24 h in contact with the different plant extracts (E) in PDB at 25 °C.

E	Concentration (*v*/*v*)	*A. niger* Log(UFC/mL)	*P. expansum* Log(UFC/mL)
Control	-	4.36 ± 0.10 ^a^	4.56 ± 0.09 ^a,b^
Echinacea extract	1%	4.35 ± 0.16 ^a^	4.7 ± 0.4 ^b^
	10%	4.44 ± 0.12 ^a^	4.60 ± 0.10 ^a,b^
Horsetail extract	1%	4.64 ± 0.07 ^b^	4.64 ± 0.02 ^a,b^
	10%	4.72 ± 0.02 ^b^	4.9 ± 0.2 ^c^
Liquid smoke	1%	4.53 ± 0.05 ^a,b^	4.56 ± 0.02 ^a^
	10%	5.04 ± 0.22 ^c^	5.252 ± 0.002 ^d^
Neem oil	1%	4.35 ± 0.10 ^a^	4.34 ± 0.06 ^e^
	10%	0 ^d^	0 ^f^

^a, b, c, d, e, f^ different letters in the same column indicate significant differences among formulations (*p* < 0.05).

**Figure 1:**
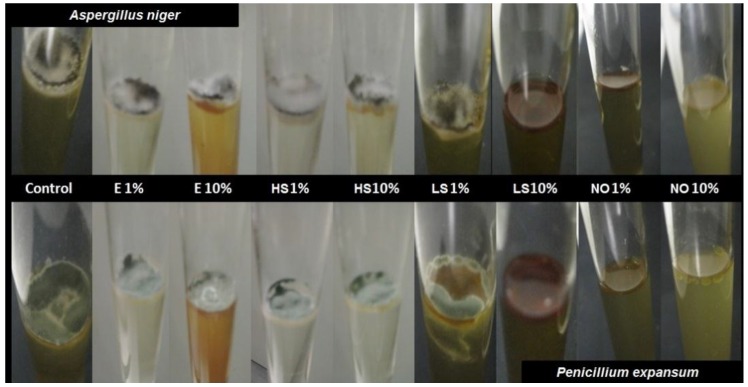
Photographs of natural extracts (1%wt and 10%wt) in PDB broth, inoculated with both fungi, *A. niger* and *P. expansum*, incubated for one week at 25 °C. E: echinacea extract; HS: horsetail; LS: liquid smoke; NO: neem oil.

The assay tubes were also subjected to a qualitative analysis after the incubation for one week at 25 °C. As can be observed in Figure 1, for tubes containing different extracts, the sporulation of both fungi occurred at surface level due to the vital necessity of the oxygen for fungi growth. Only samples containing 10% concentration of liquid smoke or neem oil at 1 and 10% showed no fungi sporulation, regardless of the fungi genera. These results partially agree with data of the viable cell counts commented on above, where only neem oil at 10% inhibited the growth of both fungi, *A. Niger* and *P. expansum*, showing fungicidal activity after 24 h of contact. This result and those previously reported [17,40,41] justify the interest in neem oil extracts as an active additive for obtaining bioactive films. According to the lack of notable antifungal activity of the rest of tested extracts, active films were formulated with neem oil and with oregano essential oil with proved antimicrobial activity [19,22].

### 3.2. Physical Characterization of Bioactive Films

Uncontrolled migration of water is generally recognized as one of the biggest problems during food storage [42]. Food coating or packaging can control or slow down this process if the water sensitivity and barrier capacity of packaging or coating materials are adequate. So, these properties are relevant in defining the functionality of the materials. Moisture content and water vapour permeability of the obtained films were characterized in films conditioned at 53% RH for 1 and 5 weeks at 25 °C and are shown in Table 2.

**Table 2:** Moisture content (MC), water vapour permeability (WVP), internal transmittance at 450 nm (Ti) and gloss at 60° incidence angle of blend control and oil composite films.

FILMS	MC (%)		WVP (gmm/sm^2^kPa)		Ti—λ = 450 nm (%)		Gloss (60°)	
	1W	5W	1W	5W	1W	5W	1W	5W
S-PVA	6.3 ± 0.5 ^a,1^	7.9 ± 0.3 ^a,2^	4.39 ± 0.14 ^a,b,1^	5.1 ± 0.8 ^a,1^	86.2 ± 0.6 ^a,1^	85.9 ± 0.6 ^a,1^	13.2 ± 1.6 ^a,b,1^	12.9 ± 1.2 ^a,1^
S-PVA-1OEO	4.4 ± 0.2 ^b,1^	8.3 ± 0.2 ^b,2^	4.6 ± 0.6 ^b,c,1^	5.2 ± 0.5 ^a,1^	84.5 ± 0.7 ^c,1^	84.5 ± 0.7 ^c,1^	14.2 ± 1.6 ^a,1^	13.5 ± 0.3 ^a,1^
S-PVA-2OEO	4.33 ± 0.13 ^b,1^	7.66 ± 0.11 ^a,2^	4.9 ± 0.5 ^c,1^	4.7 ± 0.3 ^a,b,1^	81.6 ± 0.2 ^b,1^	79.5 ± 0.2 ^b,1^	12.3 ± 1.2 ^b,1^	10.7 ± 0.8 ^b,1^
S-PVA-1NO	4.5 ± 0.3 ^b,1^	7.0 ± 0.3 ^c,2^	3.7 ± 0.3 ^a,1^	3.6 ± 0.8 ^b,c,1^	79.5 ± 0.8 ^e,1^	77.9 ± 0.6 ^e,1^	12 ± 3 ^b,1^	11 ± 3 ^b,1^
S-PVA-2NO	6.70 ± 0.13 ^a,1^	6.69 ± 0.14 ^d,1^	4.1 ± 0.4 ^a,b,1^	3.9 ± 0.8 ^c,1^	74.3 ± 0.8 ^d,1^	71.2 ± 1.4 ^d,1^	8.5 ± 0.9 ^c,1^	7. 9 ± 0.3 ^c,1^

^a, b, c, d^ different letter in the same column indicate significant differences among formulations (*p* < 0.05). ^1,2^ different number in the same file indicate significant differences among storage times (*p* < 0.05).

The moisture content of the S-PVA blend films was 6.3 and 7.9% after 1 and 5 weeks of storage, respectively, which indicates that equilibrium was not reached after 1 week. These values agree with those previously reported by Cano *et al.* [33] for the same type of films. Films containing bioactive substances showed lower moisture contents after 1 storage week, but no notable differences were observed among water sorption capacity of the films after 5 storage weeks. This indicates that the more hydrophobic nature of active films slow down the water sorption kinetics but did not notably modify the equilibrium values. Films with the highest content of neem oil (S-PVA-2NO) reached the equilibrium value after 1 storage week. The different behavior of the films could be related with the specific interactions between the oil components and the matrix depending on the concentration [19,23]. Oil components could be linked to hydroxyl groups available in starch and PVA limiting polymer–water interactions, by hydrogen bonding, thus resulting in a decrease of the film moisturizing rate [43].

WVP value for S-PVA films was similar to that previously reported by Cano *et al.* [33] for the same type of films and no significant changes occurred in this value due to the storage time. Incorporation of oils to the films did not provoke notable changes in the WVP values which were also constant during storage. So, the structural changes introduced in the polymer matrices by oils did not suppose significant changes in their water vapor barrier capacity.

The film thickness values ranged between 64 and 88 μm and were slightly influenced by the oil incorporation; no significant effect was observed for neem oil, but incorporation of oregano essential oil gave rise to slightly thicker films (83 ± 13 μm against 70 ± 14 μm for the rest of the films) despite the constant value of the solid surface concentration used [44]. This increase in thickness can be attributed to a less compact polymer matrix due to the weakening of the interchain forces provoked by the interactions of the essential oil compounds with the polymer chains. Similar behavior was described by Zivanovic *et al.* [21] and Benavides *et al.* [45] for oregano essential oil incorporated to chitosan and alginate matrices.

**Figure 2:**
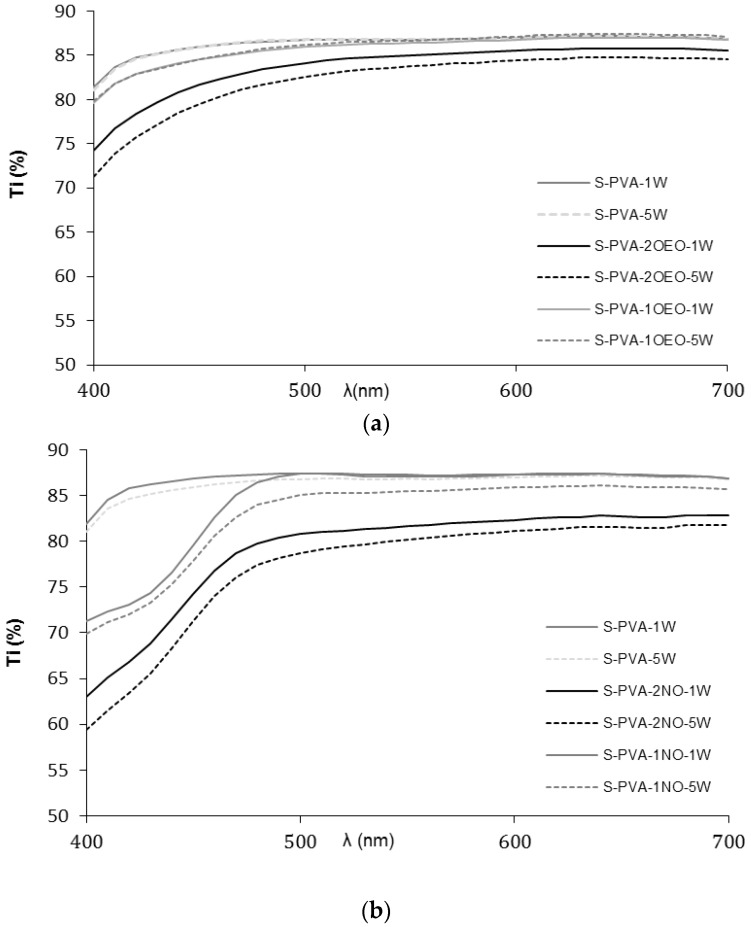
Internal transmittance of control and composite films: (**a**) films containing oregano essential oil (OEO) and (**b**) films containing neem oil (NO) after one (1W) and five weeks (5W) of storage.

The gloss and transparency of the films are relevant properties of coatings, since they have a direct impact on the appearance of the coated product [34]. Figure 2 shows the internal transmittance spectra in the visible light range (400–700 nm) of the films where the highest transparency was observed for S-PVA blend films. Films containing oils exhibited lower transparency due to the presence of a lipid dispersed phase into the polymer matrix, which promotes light dispersion, as has been previously observed by several authors [46,47]. This behavior has also been reported for films containing both neem and oregano oils [10,48,49]. Table 2 shows the values of Ti at 450 nm, where the largest difference among the films was found. S-PVA films were the most transparent with Ti values around 86% according to previous studies [33]. Ti values decreased in line with the ratio of dispersed lipid; the higher the oil ratio, the lower the film transparency, due to the promotion of light dispersion by the dispersed phase.

In general, Ti slightly decreased during the storage time, which could be attributed to an increase in the film compactness of the polymer matrices in line with the progressive chain aggregation during storage [33]. It is noticeable that neem oil gave rise to more opaque films, especially in the low wave length range, which is due to contribution of the selective light absorption of neem oil components. In this sense, the addition of oils to the films improved their light barrier property.

As concerns film gloss, Table 2 shows the values at 60° incidence angle. In general, gloss of films was significantly affected by the amount of oil in the matrix; the higher the content, the lower the gloss values. No significant changes (*p* > 0.05) in gloss occurred during storage although a decreasing tendency was observed. Gloss is related to the surface roughness of the films [46,50] and, in this sense, oil incorporation usually enhanced the presence of surface irregularities due to the flocculation and creaming of oil droplets during the film drying step and their accumulation on the film surface. Likewise, an excessive creaming could imply coalescence at surface level and the formation of a lipid layer at the top of the film. Exceptionally, S-PVA-1OEO films were glossier than other films containing oils, with values similar to the control film (S-PVA). This suggests that the oil droplets at this oil concentration are well stabilized in the film forming emulsion and no notable flocculation and creaming occurred during the film drying step.

Table 3 shows the mechanical parameters usually used to describe the film mechanical behavior: elastic modulus (EM), tensile strength (TS) and percentage of elongation (E) at break. This behavior is strongly dependent on the microstructural features of the films. TS is the maximum tensile stress that the film can sustain without break, E % is the maximum change in length before breaking, and the elastic modulus (EM) quantifies the film stiffness. Values of mechanical properties obtained for S-PVA films agreed with those previously found by Cano *et al.* [33] for similar S-PVA blend films. The mechanical properties of composite films were strongly affected by the oil concentration and storage time.

In general, films containing oil showed poorer mechanical performance than control films, especially when the highest oil concentration was added: lower values of EM and mechanical resistance (TS) and, for the highest oil content, lower extensibility. This is typical behavior when heterogeneities (oil droplets) are introduced in the matrix structure due to the lack of miscibility of components, thus reducing the overall cohesion forces of the polymer networks [19,49]. In some cases, lipid incorporation into the polymer matrices led to an increase in the film extensibility, when specific lipid–polymer interactions occurred with the subsequent plasticization effect which enhanced the film’s stretchability [51].

**Table 3:** Elastic modulus (EM, MPa), tensile strength (TS, MPa) and percentage of elongation (E, %) of blend control film and oil composite films.

FILMS	EM (MPa)		TS (MPa)		E (%)	
	1W	5W	1W	5W	1W	5W
S-PVA	506 ± 63 ^a,1^	690 ± 44 ^a,2^	26.8 ± 1.4 ^a,1^	32.3 ± 1.6 ^a,2^	40 ± 4 ^a,1^	41 ± 3 ^a,1^
S-PVA-1OEO	502 ± 50 ^a,1^	329 ± 53 ^b,c,2^	26 ± 2 ^a,1^	20.6 ± 1.5 ^b,2^	42 ± 11 ^a,1^	61 ± 9 ^b,2^
S-PVA-2OEO	271 ± 49 ^b,1^	355 ± 37 ^b,1^	4.33 ± 0.13 ^b,1^	7.66 ± 0.11 ^c,1^	4.9 ± 0.5 ^b,1^	4.7 ± 0.3 ^c,1^
S-PVA-1NO	413 ± 32 ^c,1^	296 ± 11 ^c,2^	21.5 ± 1.0 ^c,1^	19 ± 2 ^d,1^	44 ± 7 ^a,1^	60 ± 17 ^b,2^
S-PVA-2NO	174 ± 14 ^d,1^	124 ± 20 ^d,1^	7.2 ± 0.6 ^d,1^	6.8 ± 0.7 ^e,1^	11 ± 2 ^b,1^	21 ± 6 ^d,2^

^a,b,c,d,e^ different letter in the same column indicates significant differences among formulations (*p* < 0.05); ^1,2^ different number in the same file indicates significant differences among storage time (*p* < 0.05).

For films with the highest oil content, tensile strength decreased with respect to S-PVA films in more than 75% of the fils, and the percentage of elongation at break was also dramatically reduced from 40% to 20% or 5% for films containing neem oil and oregano essential oil, respectively. On the contrary, Hosseini *et al.* [49] reported an increase in the plastic deformation for fish gelatin-chitosan films when the amount of oregano essential oil in the films (0.4 to 0.8%–1.2% *w*/*v*) was increased.

After five weeks of storage, S-PVA films showed an increase in their resistance to breakage as well as in their rigidity whilst no significant changes in their elongation capacity were observed. These changes can be attributed to the progressive increase in the matrix compactness in line with the progress of chain aggregation. This is promoted by means of the establishment of further interactions between both polymers, *i.e.*, the formation of oxi (-O-) groups and hydrogen bonds to some extent between the hydroxyl groups of the starch and PVA chains [33,52,53,54].

On the contrary, films containing NO and low concentration of OEO became more stretchable during storage (*p* < 0.05). This behavior suggests that oil components’ interactions with the polymer chains were progressively established, this contributing to a strong plasticization effect in the matrix and inhibiting the polymer chain aggregation. The different behavior of films with the highest ratio of the OEO could be explained by having a too high of an oil content for it be effectively entrapped in the polymer network, giving rise to a predominant effect of network weakening.

Oregano essential oil at the highest ratio imparted the poorest mechanical response to the films and, in general, better results were obtained for films containing the lowest levels of both oils (good values of rigidity and resistance and the greatest stretchability). These films exhibit similar mechanical parameters to some commercial plastics which are very flexible and resistant, such as those found by Cano *et al.* [33] for low density polyethylene (LDPE) bags with similar thickness (EM = 370 MPa, TS = 27 MPa and %E = 40%).

### 3.3. Antibacterial Activity of Composite Films

**Figure 3:**
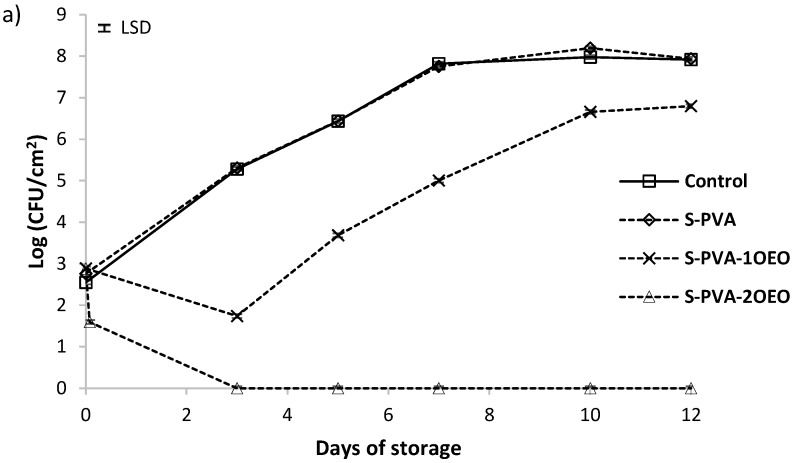

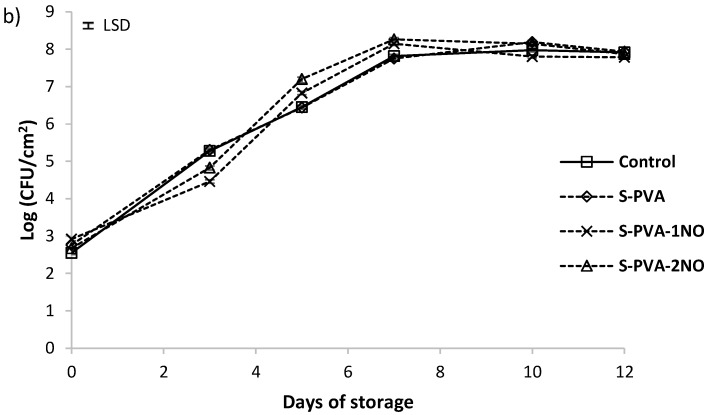
Population viability of *Listeria innocua* in TSA-NaCl medium at 10 °C (**a**) films with and without oregano essential oil (OEO) and (**b**) films with and without neem oil (NO). Mean values for each incubation period and 95% LSD interval are included in the plot. (LSD = ±0.05).

**Figure 4:**
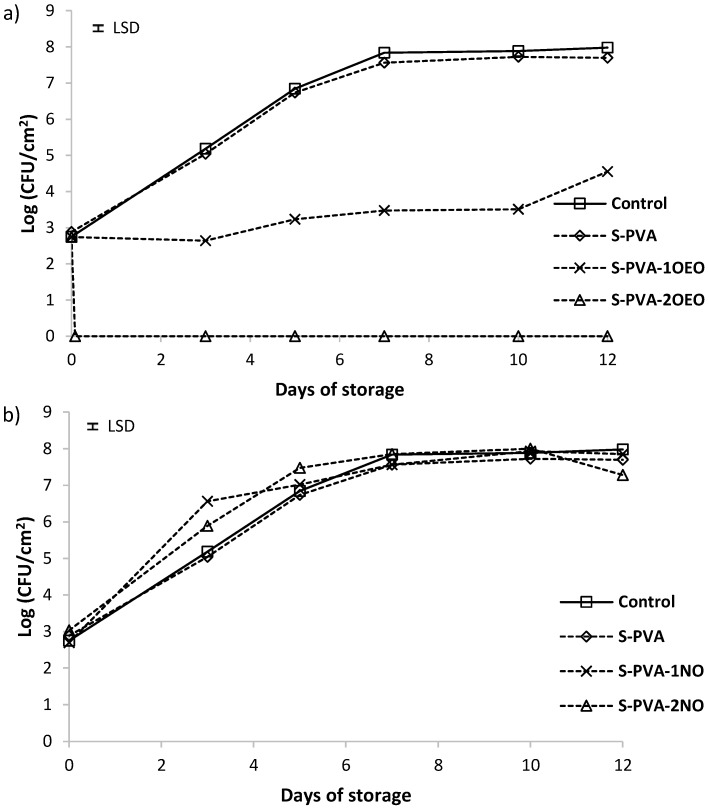
Population viability of *Escherichia coli* in TSA-NaCl medium at 10 °C (**a**) films with and without oregano essential oil (OEO) and (**b**) films with and without neem oil (NO). Mean values for each incubation period and 95% LSD interval are included in the plot. (LSD = ±0.07).

Population viability of *Listeria innocua* and *Escherichia coli* in control plates and in plates coated with the different films is shown in Figure 3 and Figure 4, respectively. For both bacteria, population increased from 2.5 to 8 logs UFC/cm^2^ at the end of the storage period. No significant antimicrobial activity (about 1 log reduction) was observed for S-PVA films without oil throughout the incubation time at 10 °C, where the bacterial growth was very similar to that of control plates (without film).

The incorporation of neem oil at both ratios (Figure 3b and Figure 4b) did not improve the antimicrobial properties of S-PVA films, even more, neem oil seems to promote the early growth of bacteria during the first storage period, showing a larger population than S-PVA films. On the other hand, the incorporation of oregano essential oil at the two proportions (Figure 3a and Figure 4a) promoted antimicrobial properties in S-PVA films, showing a significant antibacterial activity since the first storage time. This effect depended on the essential oil concentration. At the highest oil concentration (S-PVA-2OEO), films showed bactericidal effect just two hours after the plate coating. Meanwhile, the lowest concentration of this oil in the films (sample S-PVA-1OEO) only slowed down the bacterial growth during the incubation period.

Different authors reported that films containing essential oils are more effective against Gram-positive than against Gram-negative bacteria [19,26,34,49]. However, oregano essential oil gave rise to S-PVA active films, with an antibacterial and bactericidal effect particularly stronger against Gram-negative bacteria, as can be observed in Figure 3a and Figure 4a. Similar results were also reported by Muriel-Galet *et al.* [55] for the oregano essential oil embedded in ethylene–vinyl alcohol copolymer (EVOH) films. This behavior has been previously described as a specific action of the OEO compounds [22,56,57]. In this sense, the main components of OEO (carvacrol and thymol) are able to disintegrate the outer membrane of Gram-negative bacteria, releasing lipopolysaccharides (LPS) and increasing the permeability of the cytoplasmic membrane to ATP.

### 3.4. Antifungal Activity of Composite Films

**Figure 5:**
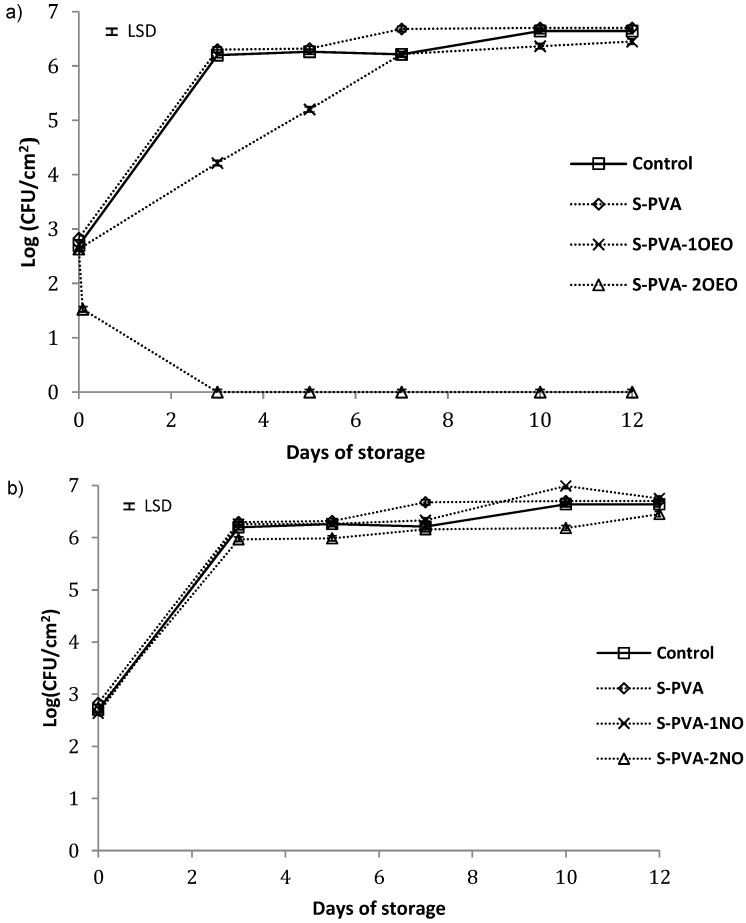
Population viability of *Penicillium expansum* on PDA medium incubated at 25 °C. (**a**) Films with and without oregano essential oil (OEO) and (**b**) films with and without neem oil (NO). Mean values for each incubation period and 95% LSD interval are included in the plot. (LSD = ±0.05).

**Figure 6:**
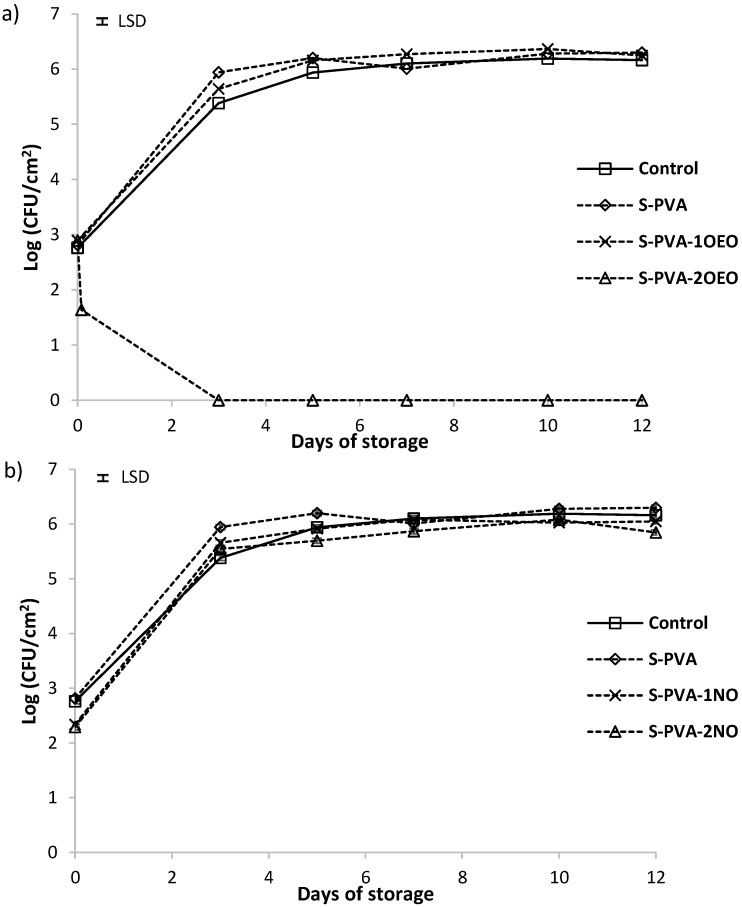
Population viability of *Aspergillus Niger* on PDA medium incubated at 25 °C. (**a**) Films with and without oregano essential oil (OEO) and (**b**) films with and without neem oil (NO). Mean values for each incubation period and 95% LSD interval are included in the plot. (LSD = ±0.05).

The possible antifungal effect of developed S-PVA films against *Penicillium expansum* and *Aspergillus niger* was analyzed at 25 °C. Figure 5 and Figure 6 show the cell viability level, for 10^5^ spores/mL initial population, of *P. expansum* and *A. niger*, respectively. For both fungi, population increased from 2.5 to 6.5 log UFC/cm^2^ at the end of the incubation period in all cases, except for the films containing the highest amount of OEO. Control plates (without film) and those coated with S-PVA films (without oil) showed a similar trend without antifungal activity.

The incorporation of neem oil at both ratios did not improve the antifungal activity of the S-PVA films despite the activity detected for this oil in the screening test. This could be due to the strong entrapment of the oil compounds in the film structure, which inhibits their diffusion to the film surface where fungal growth occurs. Neem oil is mainly constituted (about 87%) by long chain fatty acids (oleic, stearic, and palmitic acids) [58], which can strongly interact with hydroxyl groups of the polymers, thus limiting their diffusion to the film surface and so the exhibition of the antifungal effects.

The presence of oregano essential oil in the films affected the fungal growth of both fungi genera, depending on its concentration in the matrix, as previously observed by Sánchez-González *et al.*, 2010 tea tree essential oil embedded in chitosan films. At the lowest OEO level (S-PVA-1OEO), no antifungal effect was observed against *A. niger* whilst the growth of *P. expansum* was inhibited throughout the first seven incubation days (2 log reduction with respect to the control film). Nevertheless, after seven days of storage, no significance difference in the fungus growth with respect to the control film was observed. This behavior could be due to the losses of active compounds over time, maintaining adequate concentration of active films on the agar medium surface till seven days. After this time, the low availability of the active compounds on the surface led to the growth of fungi due to the prevalent contamination [34,39].

At the highest oregano oil concentration (S-PVA-2OEO), fungicide effect was observed just after two hours of plate coating. No growth of fungi during the storage period was observed, thus indicating the lethal fungicidal effect of the oil components (carvacrol and thymol) in agreement with those reported by other authors [55].

From the obtained results, it can be deduced that OEO is highly effective in limiting the growth of gram-positive and gram-negative bacteria embedded in the S-PVA films, even at very low concentrations, and was effective to control fungus at moderate ratios. The higher effect on the bacteria growth can be attributed to the simpler cellular wall of this microorganism as compared to the fungal cell walls.

## 4. Conclusions

Composite films based on starch-PVA blends, containing potentially antimicrobial oils, exhibit antibacterial (*L. innocua* and *E. coli*) and antifungal (*A. niger* and *P. expansum*) properties when they contain oregano essential oil (OEO), whereas active neem oil did not impart these properties to the matrix. Antibacterial activity occurred at low OEO concentration (6.7% in the dried matrix), while antifungal effect required higher doses of oil in the films. Incorporation of oils did not notably affect the water sorption capacity and water vapor barrier properties of S-PVA films, but reduced their transparency and gloss, especially at the highest concentration (22% in the dried matrix). Mechanical performance of the S-PVA films was also modified by incorporation of oils but this was only relevant at the highest oil ratios. For the lowest oil concentration, the mechanical properties of the S-PVA composites were in the range of those of some commercially available bags, becoming slightly more plasticized after five weeks of storage. Among developed composite films, those containing 6.7% of OEO exhibited the best physical properties, without significant differences with respect to the S-PVA matrix, while also exhibiting antibacterial activity. So, these active films containing oregano essential oil represent a novel and good alternative for use in food packaging. These films could be used to extend the shelf-life of products such as bread and cheese, and as a coating material in fruits such as oranges, lemon and mangos by using only natural compounds with antimicrobial activity.
